# Correlation between Pathological Characteristics and Young's Modulus Value of Spastic Gastrocnemius in a Spinal Cord Injury Rat Model

**DOI:** 10.1155/2017/5387948

**Published:** 2017-12-27

**Authors:** Li Jiang, Yu-jue Wang, Qiao-yuan Wang, Qing Wang, Xiao-mei Wei, Na Li, Wei-ping Guo, Zu-lin Dou

**Affiliations:** ^1^Department of Rehabilitation, The 3rd Affiliated Hospital of Sun Yat-sen University, Guangzhou, China; ^2^Department of Ultrasound, The 3rd Affiliated Hospital of Sun Yat-sen University, Guangzhou, China; ^3^Institute of Medical Information, School of Biomedical Engineering, Southern Medical University, Guangzhou, China; ^4^Department of Gastroenterology Surgery, The 3rd Affiliated Hospital of Sun Yat-sen University, Guangzhou, China

## Abstract

The goal of the present study were (1) to investigate the pathological characteristics of gastrocnemius muscle (GM) and quantitatively assess GM tissue stiffness in rat models with spinal cord injury (SCI) and (2) to explore the correlation between pathological characteristics changes and Young's modulus value of GM. 24 Sprague Dawley male rats were allocated into normal control groups and SCI model subgroups, respectively. GM stiffness was assessed with shear wave sonoelastography technology. All GMs were further analyzed by pathological examinations. GM weights were decreased, the ratio of type I fibers was decreased, and the ratio of type II fibers was increased in the GM in the model group. MyHC-I was decreased, while MyHC-II was increased according to the electrophoretic analysis in model subgroups. The elastic modulus value of GM was increased in the model group. A significant negative correlation was found between Young's modulus value of GM and the ratio of type I fibers of GM in model subgroup. Our studies showed that the stiffness of GM is correlated with pathological characteristics during the initial stages of SCI in rats. We also identified shear wave sonoelastography technology as a useful tool to assess GM stiffness in SCI rat models.

## 1. Introduction

Spasticity is a common disorder in patients with injury of the brain and spinal cord. Severe spasticity impacts patient limb function and subsequently their daily life. Previous studies have shown that spastic skeletal muscle secondary structures changes (such as shortening of muscle fibers, increased connective tissue, and adipose tissue) can modify certain biomechanical properties, such as increased stiffness levels [1, 2]. Understanding the pathophysiology of spasticity may provide important clues to its treatment. According to studies on the pathophysiology of spasticity, exaggerated reflexes and secondary changes in mechanical muscle fibers properties have a major role in spastic movement disorder [3]. Clinically, spasticity is associated with increased muscle tone, stiffness, and eventual joint contractures [4]. Spastic muscle stiffness can reflect the level of spasticity. The assessment of spastic muscle stiffness is conductive to the development of personalized spasticity treatment strategy but also helpful to study the effectiveness of the therapeutic intervention.

Clinicians often judge the changes in muscle hardness by passively pulling the spastic limbs and touching the spastic muscle, and the assessment results are greatly influenced by the subjective factors from the examiner. It is essential to have an objective, quantifiable method of measuring spasticity muscle stiffness. Sonoelastography can directly detect Young's modulus value of biological tissue to determine its elasticity. On sonoelastographic images, a relaxed muscle structure will appear mostly soft (green blue), while contracted or degenerated muscle fiber will appear hard (red) [5]. This imaging method further promotes the comparison of elastic properties under various physiological conditions [6]. Despite its known advantages, this technique has been applied for the evaluation of spastic muscles stiffness following UMN injury [5, 7].

Muscle biopsy is the most prevalent type of analysis of spastic muscle tissue. Sectioned muscles from patients with spasticity show abnormalities such as increased variability in fiber size. Variability in fiber size (i.e., large and small fibers within the same muscle) is characteristic of numerous neuromuscular disorders and not specific to spasticity [2]. Some biopsy studies report an increased percentage of type I fiber in muscle from patients with spasticity and fewer report an increased percentage of Type II fiber. There is no general agreement on muscles issue which must be due, in part, to the sampling problems. Animal models of spasticity have allowed for the elucidation of possible mechanisms and the evaluation of potential therapeutic interventions for these serious clinical problems. Only complete spinal transection at the thoracic level in animal models would duplicate completely and permanently what is seen in humans after SCI. The rat models with SCI have been used as experimental subjects for spasticity muscles in most laboratories [8, 9].

In a previous study [10], we reported changes in the pathological characteristics and Young's modulus values of GM in rats with completed spinal cord injury (SCI). This brought up the question of whether there is a correlation between these pathological GM characteristics and Young's modulus value in SCI model rats. Therefore, in this study, we used SCI rat models to explore the pathological changes and Young's modulus values of GM in rats with SCI at different time points, and the correlation analysis was used to determine whether the pathological characteristics were correlated with Young's modulus value. The results of the study will reveal the relevant factors of spastic muscles and help to find the treatment strategies to reduce muscle stiffness.

## 2. Materials and Methods 

### 2.1. Materials

Forty-two Sprague Dawley male rats (260–280 g) were divided into the control group (6 rats) and the SCI model group (36 rats). The rats in the SCI model group were randomly divided into the 2 w, 4 w, and 12 w subgroups (12 rats per group). This study was approved by the Sun Yat-sen University Center for Ethical Review (approval number: IACUC-20140201).

### 2.2. Methods

#### 2.2.1. Model Preparation

The rats were anesthetized by the abdominal injection of 10% chloral hydrate solution at a dose of 0.35 ml/100 g body weight [8, 9]. Following anesthetization, the rats were fastened to a sterile operating table and the spinal cord was completely severed at the T10 level before a gelatin sponge was placed in severed space (3 × 2 × 2 mm). We then sutured the muscle, fascia, and skin of the rats. Following principles from a previous study, we took measures to prevent postoperative infection and used an artificial extruding bladder to assist with micturition [8].

#### 2.2.2. Muscle Tone and Mobility Behavioral Assessment

Plantar flexor muscle tone was assessed using the Modified Ashworth Scale (MAS). Mobility behavior was assessed using the Basso, Beattie, and Bresnahan Locomotor Rating Scale (BBB Scale) [11]. The assessors were double-blinded to the groups.

#### 2.2.3. Supersonic Sonoelastography

An AixPlorer ultrasonic scanner (Supersonic Imagine, Aix en Provence, France), coupled with a linear transducer array (4–15 MHz, SuperLinear 15-4, Vermon, Tours, France), was used in the present study. The scanner was set at the supersonic shear imaging (SSI) mode (musculoskeletal preset). SSI operates on a transient elastography principle. It produces elastography images based on the combination of a radiation force and an ultrafast ultrasound acquisition imaging system capable of capturing in real time the propagation of the resulting shear waves. The elastic modulus can be calculated from the velocity of the propagating wave when a faster velocity indicates a greater elastic modulus. Therefore, the elastic modulus can be calculated by measuring the propagation of shear waves. A light touch on the skin with the ultrasound probe is suggested by the manufacturer and a quantitative elasticity map can be computed from the system within a few milliseconds [12].

**Figure 1:**
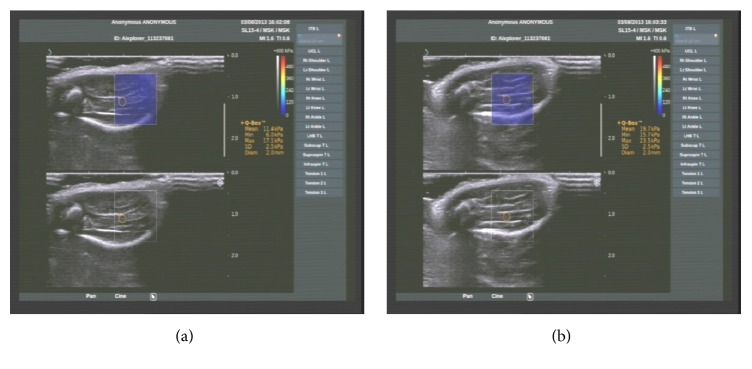
Image of sonoelastography ((a) ankle flexion; (b) ankle extension).

Measurement positions included ankle flexion (0°) and extension (−90°). Rat calf skin hair was removed, and each rat was laid on its left side with the right lower limb placed on a platform (hip and knee 90° flexion). Next, the probe was lightly placed on the skin above the GM, ensuring that the long axis of the probe was perpendicular to the tibia. The ROI was set to 10 × 10 mm, and the depth was set to approximately 0.5–1.0 cm. The probe was then rotated 90° to make the long axis parallel to the tibia. The two-dimensional gray-scale and elastic images were simultaneously observed with double real-time imaging. The elasticity imaging mode was then employed, followed by the application of the acoustic radiation pulse and detection of shear wave after the muscle elasticity image was stable. The image was then captured when the Q-BOX function measuring Young's modulus value (kPa) was initiated (Figure 1). Each image was captured five times for each plane measurement and all plane measurements were averaged.

#### 2.2.4. Type of Muscle Fiber and Myosin Heavy Chain Isoform Quantification

In order to obtain the GM subsistence values, Type I, Type IIa, and Type IIb muscle fibers were counted in ATPase stain by Image J. Next, the GM Type I, IIa, IIb, and IIx myosin heavy chain (MyHC) data was analyzed with electrophoresis. The percentage proportions of MHC isoforms in the analyzed muscles were estimated by comparing the degree of staining intensity with Coomassie brilliant blue [13]. Quantity one software was applied for data analysis.

#### 2.2.5. Statistical Analysis by SPSS 19.0 Software

Measurement data were described as the difference in means. One-way analysis of variance (ANOVA) was used to analyze differences among groups. The least significant difference *t*-test was used to analyze data with constant variances. Dunnett's *T*3 test was used to analyze data without constant variances. Pearson's correlation analysis was applied to the correlation between Young's modulus value and the type ration. *P* < 0.05 was considered to indicate a statistically significant difference.

## 3. Results

After removing the overweight and underweight rats, six rats were included in each subgroup.

### 3.1. General Assessment

The overall weight of the SCI rats in the 2 w subgroup was lower than those of the control and 12 w subgroups (*P* < 0.05). However, the difference between the weight of the SCI rats in the 2 w and 4 w subgroups was not significant. The weights of the SCI rats in the 4 w and 12 w subgroups were higher than those of the control group, but the difference was not statistically significant. The GM weight of the rats in the 2 w subgroup was the lightest. The weights of the GMs were higher in the 4 w and 12 w subgroups compared to the control group (*P* < 0.05). The BBB scores of the rats in the 2 w subgroup were the lowest. Interestingly, the BBB scores of the rats in the 2 w and 4 w subgroups were significantly lower than in the 12 w subgroup (*P* < 0.05). The MAS scores of the three subgroups were higher than that of the control group (*P* < 0.05) (Table 1).

### 3.2. Young's Modulus Value

All of the sonoelastography images were acquired from the right GM of rats. Measurement positions included ankle flexion (0°) and extension (−90°). In the control group, Young's modulus value of the ankle flexion was higher than the ankle extension (*t* = 1.93, *P* = 0.00). In each SCI subgroup, Young's modulus value of the ankle flexion was higher than the ankle extension (*P* < 0.05).

Compared with the control group, Young's modulus value gradually increased with time in the area under the ankle flexion position (*F* = 29.78, *P* < 0.01) in the subgroups. Young's modulus value for the 2 w subgroup was significantly lower than that for the 4 w and 12 w subgroups, and the 4 w subgroup value was significantly lower than the 12 w subgroup. In the ankle extension position, Young's modulus value of three SCI subgroups was significantly lower than that of the control group (*P* < 0.05). However, the results of the three SCI subgroups reached statistical significance when compared with each other (Table 2).

### 3.3. ATPase Stain

The proportion of Type I fiber in the three SCI subgroups was significantly lower than in the control group (*F* = 9.99, *P* < 0.01). The proportion of Type IIa muscle fiber in the three SCI subgroups was significantly higher than that of the control group (*F* = 5.96, *P* < 0.01). However, the results of Type I and Type IIa fiber in the three SCI subgroups were not statistically different when compared to each other. The difference in the proportion of the Type IIb fiber from the three SCI subgroups was not statistically significant when compared to the control group (*F* = 2.63, *P* = 0.08) (Table 3).

**Figure 2:**
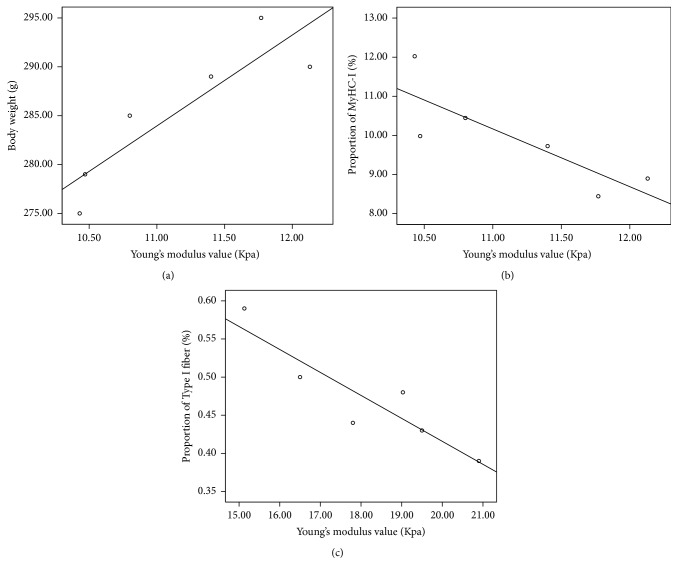
The correlation between Young's modulus value and weight (a), proportion of MyHC-I (b), and proportion of Type I muscle fiber (c) in rats model. (a), (b) Ankle flexion; (c) ankle extension.

The rat GMs included four types of MyHC, namely, MyHC-I, MyHC-IIa, MyHC-IIb, and MyHC-IIx, according to the electrophoretic analysis (Figure 2). In the control group, the proportions of MyHC-I, MyHC-IIa, MyHC-IIb, and MyHC-IIx were 9.91 ± 1.26%, 5.55 ± 0.18%, 45.34 ± 1.95%, and 39.20 ± 1.47%, respectively. In the SCI group, the proportion of MyHC-I decreased significantly compared with the control group (*F* = 50.49, *P* < 0.01) and, in the 4 w and 12 w subgroups, was significantly lower than that observed in the 2 w subgroup (*P* < 0.05). Furthermore, the proportion of MyHC-I in the 12 w subgroup was significantly lower than that in the 4 w subgroup (*P* < 0.05). The proportions of MyHC-IIa in all three SCI subgroups were higher than the control group. However, the three SCI subgroups were statistically significantly different in the proportion of MyHC-IIa when compared to each other (*P* < 0.05). The proportions of MyHC-IIx in all of the SCI subgroups were higher than that observed in the control group; however, there was no statistically significant difference when compared with each other (*P* > 0.05). Nevertheless, the proportion of MyHC-IIb in the three SCI subgroups was not significantly different when compared with the control group (*P* > 0.05) (Table 4).

### 3.4. Correlation Analysis

**Figure 3:**
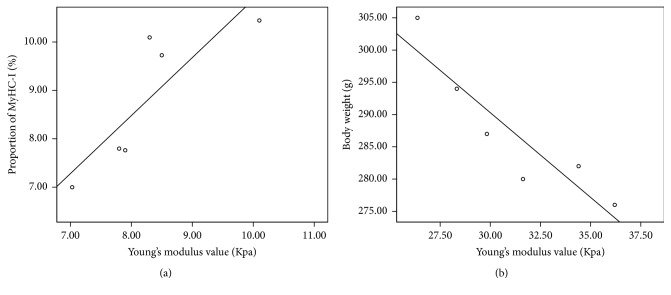
The correlation between Young's modulus value and the proportion of MyHC-I (a) and weight (b). (a) Two-week subgroup, ankle flexion. (b) Four-week subgroup, ankle extension.

In the control group, when the rats were in the ankle flexion state, Young's modulus value was positively correlated with body weight (*r* = 0.89, *P* = 0.02; Figure 2(a)) and negatively correlated with the proportion of MyHC-I (*r* = −0.83, *P* = 0.04; Figure 2(b)). In the 2 w subgroup, Young's modulus value was negatively correlated with the proportion of MyHC-I (*r* = −0.85, *P* = 0.03; Figure 3(a)). In the control group, when the rats were in the ankle extension state, Young's modulus value was negatively correlated with the proportion of Type I fiber (*r* = −0.91, *P* = 0.01; Figure 2(c)). In the 4 w subgroup, Young's modulus value was found to be negatively correlated with body weight (*r* = −0.92, *P* = 0.01, Figure 3(b)).

## 4. Discussion

The results of the comparative analysis in this study showed that each subgroup displayed a significant decrease in body weight and GM weight, which was similar to the results from previous investigations [14, 15]. The main reason for this could be a combination of the denervation, atrophy, and paralysis of the muscle below the injury site. Furthermore, reports show that a decline in muscle mass following injury was associated with poor appetite during the acute stage, after surgery [14]. As time progressed in the present study, the general condition of the muscle in the SCI group became more stable, the SCI was gradually restored to normal conditions, and animal weight increased. In addition, the function of the lower limb movement gradually recovered with time. The peak MAS appeared in the 2 w subgroup rats. A decrease in MAS, however, was apparent in the 4 w subgroup. In the 12 w subgroup, the MAS value was lower than was observed in the 2 w and 4 w subgroups but higher when compared to the control group. The BBB score was lowest in the 2 w subgroup. This group exhibited incomplete combined movement in the hip and knee joint. Incomplete flexion and extension movement of the ankle joint appeared in rats of the 4-w subgroup. At 12 weeks after injury, the BBB score increased to approximately 7-8 scores [15]. The recovery of rats in each subgroup displayed individual differences, with the 2 w subgroup being the worst and the 12 w subgroup being the best of the SCI rats. In this respect, the results are similar to previous studies. In this study, the SCI completely debilitated the rats, which were never able to fully recover to normal conditions.

The GM of rats includes four types of muscle fibers: Type I, Type IIa, Type IIb, and Type IIx. Muscle fiber Type I, Type IIa, and Type IIb can be distinguished by ATPase staining [15]. The proportion of Type I muscle fiber significantly decreased following injury. In contrast, the proportion of Type II muscle fiber significantly increased following injury. The observed trend of change is similar to those reported in previous studies [16]. Electrophoresis showed that the proportion of MyHC-I, MyHC-IIa, and MyHC-IIx were significantly modified, similar to previous studies [17]. Among these changes, the increased Type IIa muscle fiber was the most obvious change. At 12 weeks, we observed a 60% Type IIa increase, which was significantly higher than the proportion of Type I muscle fibers (20%). The proportion of Type IIb muscle fiber showed a decreasing trend but did not reach statistical significance when compared with the control group. These changes appear to be strongly related to SCI and reduced movement. Moreover, the transition to a different type of muscle fiber has been shown to be caused by the ability of mutual transformation between different types of skeletal muscle fiber, namely, plasticity. Studies have confirmed that electrical stimulation and weightlessness can induce the transformation and its plasticity is related to the state of the muscle fibers [18]. Previous studies on SCI rats found that Type I GM muscle fibers can transform into Type II muscle fibers [10, 19]. Nevertheless, the shift between muscle fiber types can be reversed through the correct amount of electrical stimulation. It is widely known that the physiological function of each muscle fiber is different. Type I muscle fibers produce small, extended tension. However, Type II muscle fibers produce long fibers with a short tension time. Skeletal muscle constitutes different fibers and produces different types of tension [18]. Modifications to muscle fiber types influence the physiological function and biomechanical characteristics of skeletal muscle, such as shortened relaxation time and fatigue, also influencing the normal muscle contraction function.

Clinical studies have confirmed that the elastic modulus value is greater when muscles are in contraction compared to their state in relaxation. Thus, the elastic modulus value can tell the state of the muscle [6]. At different ankle positions, the contractive condition of GM was found to also be different. For the more comprehensive evaluation of the hardness characteristics of GM spasm in SCI rats, this study included two measuring positions: ankle extension (−90°) and flexion (0°). In the ankle extension position, Young's modulus value for the GM in each subgroup of SCI rats was less than that in the control group. The minimum value appeared at the 2nd week and was improved at the 12th week. A reason for this result may be that the muscle had undergone paralysis. At the 2nd week, the muscle paralysis was at its most severe. Paralysis recovers over time; therefore, by the 12th week, GM had regained the initiative movement. In the ankle flexion position, Young's modulus value of SCI rats in each subgroup was significantly higher than that of the control rats. Previous studies showed that the hardness of spasmodic muscle is significantly higher than normal controls [6, 7]. In addition, we found that Young's modulus value in the state of ankle flexion was significantly higher than the value in the state of ankle extension for each SCI subgroup. We believed that the difference observed between the two conditions was caused by the abnormal state of joints after SCI. After lower limb paralysis, the ankle state changes from extension to flexion, and this change remains until normal movement is restored. Moreover, the GM stays in a tension-free condition when the ankle is in the extension state. In turn, the GM becomes tense when the ankle flexes. Therefore, the elastic modulus value increases, causing Young's modulus value to also increase. Additional causes of the degree of GM spasm include weight, fiber classification, and muscle structure change. Another factor to consider is the assessment of the hardness of the ankle flexion as a suitable position to measure the hardness of GM spasms, as found in the present study.

Based on the above results, we know that the pathological characteristics and elastic modulus dynamically change after SCI. Therefore, we posed the question of whether GM hardness is correlated with pathological change. The results of our correlative analysis showed that, for normal rats, Young's modulus value was positively correlated with the body weight observed in the control group and was negatively correlated with the proportion of MyHC-I in the ankle flexion state. Young's modulus value was negatively correlated with the proportion of Type I muscle fibers when the ankle was in the extension state. In this study, the normal rats with higher level weight own bigger mass GM than rats with lower weight and bigger mass GM appeared to be stiffer than smaller mass GM. As we know, Type I muscle fiber produces small, sustained tension, and Type II muscle fiber produces large, short-term tension. The different muscle fibers constituents result in different tension levels [18]. In addition, GM with higher proportion of MyHC-I showed lower tension and stiffness in either flexion or extension state. Furthermore, for the SCI rats, Young's modulus value was negatively correlated with the proportion of MyHC-I in the 2 w subgroup and was negatively correlated with body weight in the 4 w subgroup. We considered the change of the pathological features of GM in rats with SCI was complicated. Body weight, ration of muscle fibers, and proportion of MyHC all varied following the injury course. However, for the different time point subgroup, the influence of SCI was different. Young's modulus value is lowest in 2 w subgroups. Like 2 w rats model, we thought the increased proportion of MyHC-I is one of the causes that resulted in decreased Young's modulus value during ankle flexion in the study. For 4 w rats model, weight mass increased following rats condition improvement. GM showed more tension at flexion state in rats with lower weight, resulting in a higher Young's modulus value. During recovery, for 12 w subgroup, rats model got better and better condition, and proportion of MyHC and ratio of muscle fibers got normal; however, GM stiffness did not show any decrease. We will make more efforts to make the issue clear in the future.

Also, skeletal muscle includes an inner and outer membrane and perimysium. These compositions are the foundation of muscle elasticity. Previous studies found that rats with SCI have extracellular connective tissue modifications during recovery [2, 17]. Due to limited experimental methods, this study did not include quantitative measurements for changes in muscle composition. Future experiments will determine whether Young's modulus value is related to these pathological changes.

## 5. Conclusions 

The present study used sonoelastography to evaluate GM in rats after SCI. From these studies, we found that this imaging technique can be used in the evaluation of modifications to GM hardness in rats following SCI. We observed a series of GM dynamic changes in rats at 2, 4, and 12 weeks following complete SCI. Such a decrease in muscle weight and the change of muscle fiber composition led to the conclusion that the effects of SCI are not limited to pathological changes, but also to changes in the hardness of the GM (Young's modulus value) area affected by SCI. Modifications to the hardness of the GM in SCI rats were related to pathological changes (weight, the type of MyHC, and muscle fiber type of GM) that took place during the earlier phase following SCI. Moreover, Young's modulus value observed at the later times may be related to modifications in the extracellular connective tissue.

## Figures and Tables

**Table 1:** Results of the general assessment in different groups.

Group	Body weight (g)	GM weight (g)	BBB (score)	MAS (score)
SCI				
2 w	275.33 ± 6.42	0.93 ± 0.18	3.17 ± 0.98	1.50 ± 0.48
4 w	287.33 ± 10.65	0.98 ± 0.45	4.83 ± 0.97	1.33 ± 1.03
12 w	290.33 ± 16.21	1.24 ± 0.11	7.17 ± 1.33	0.83 ± 0.41
Control	285.50 ± 7.42	1.62 ± 0.07	21.00	0
*F*	2.11	9.70	429.72	4.67
*P*	0.13	<0.01	<0.01	0.01

**Table 2:** Results of Young's modulus value in different groups.

Group	Ankle flexion	Ankle extension	*t*	*P*
SCI				
2 w	8.27 ± 1.03	25.08 ± 2.40	−14.69	<0.01
4 w	8.94 ± 0.62	31.13 ± 3.71	−13.71	<0.01
12 w	8.33 ± 2.54	37.38 ± 5.54	−13.29	<0.01
Control	11.17 ± 0.71	18.14 ± 2.10	−7.93	<0.01
*F*	5.29	29.78	—	—
*P*	0.01	<0.01	—	—

**Table 3:** The proportion of each type of muscle fiber in different groups.

Group	Type I	Type IIa	Type IIb
SCI			
2 w	0.25 ± 0.12	0.42 ± 0.24	0.34 ± 0.12
4 w	0.19 ± 0.12	0.49 ± 0.19	0.33 ± 0.07
12 w	0.18 ± 0.10	0.61 ± 0.20	0.21 ± 0.13
Control	0.46 ± 0.10	0.15 ± 0.12	0.38 ± 0.09
*F*	9.99	5.96	2.63
*P*	<0.01	<0.01	0.08

**Table 4:** The proportion of MyHC electrophoresis results in SCI rats.

Group	MyHC-I	MyHC-IIa	MyHC-IIx	MyHC-IIb
SCI				
2 w	8.80 ± 1.45	3.68 ± 1.61	43.50 ± 3.46	44.01 ± 5.18
4 w	4.58 ± 2.26	3.38 ± 0.93	44.22 ± 3.45	47.81 ± 3.33
12 w	0.44 ± 0.25	4.25 ± 1.50	45.60 ± 2.91	49.71 ± 3.66
Control	9.91 ± 1.26	5.55 ± 0.18	39.20 ± 1.47	45.34 ± 1.95
*F*	50.49	3.85	5.31	2.81
*P*	<0.01	0.03	0.01	0.07
